# Access to community-based HIV services among transgender women in Cambodia: findings from a national survey

**DOI:** 10.1186/s12939-019-0974-6

**Published:** 2019-05-17

**Authors:** Siyan Yi, Say Sok, Srean Chhim, Pheak Chhoun, Navy Chann, Sovannary Tuot, Phalkun Mun, Marija Pantelic

**Affiliations:** 1 Saw Swee Hock School of Public Health, National University of Singapore & National University Health System, Tahir Foundation Building, 12 Science Drive 2, #10-01, Singapore, 117549 Singapore; 2 KHANA Center for Population Health Research, Phnom Penh, Cambodia; 3 Center for Global Health Research, Touro University California, Vallejo, USA; 4 Surveillance Unit, National Center for HIV/AIDS, Dermatology and STD, Phnom Penh, Cambodia; 5 FHI 360 Cambodia, Phnom Penh, Cambodia; 6 Frontline AIDS, Brighton, UK; 7 Department of Social Policy and Intervention, University of Oxford, Oxford, UK

**Keywords:** Service accessibility, Community-based services, HIV key population, Transgender people, Respondent-driven sampling, Cambodia

## Abstract

**Background:**

Globally, the prevalence of HIV among transgender women is more than 40 times higher than the prevalence in the general reproductive-age adults. They also face intersecting barriers to health, social, and legal services due to their hidden and stigmatized nature. Despite the particular needs, data regarding the access to services among transgender populations is scant globally. This study aims to identify characteristics of transgender women in Cambodia that may determine their accessibility to community-based HIV services.

**Methods:**

In the National Biological and Behavioral Survey 2016, a structured questionnaire was used for face-to-face interviews with 1375 sexually active transgender women recruited from the capital city and 12 other provinces using the Respondent-Driven Sampling method. Weighted multivariate regression analysis was conducted to explore factors associated with access to community-based HIV services.

**Results:**

The mean age of the participants was 25.8 years (SD = 7.1), and 45.0% reported having received at least one community-based HIV service in the past three months. Compared to participants who reported not having been reached by any community-based HIV programs, participants who reported having been reached by the programs were significantly more likely to reside in an urban setting (AOR = 1.41, 95% CI = 1.01–1.96), to have used gender-affirming hormones (AOR = 1.50, 95% CI = 1.17–1.92), to have been tested for HIV in the past six months (AOR = 7.42, 95% CI = 5.78–9.53), and to have been arrested by police or other authorities because of their transgender identity (AOR = 1.55, 95% CI = 1.03–2.33). Participants who reported having been reached by community-based HIV programs were significantly less likely to report being in a receptive role (AOR = 0.34, 95% CI = 0.15–0.82), to use condoms consistently with non-commercial male partners (AOR = 0.72, 95% CI = 0.55–0.94), and to perceive that their co-workers were not supportive regarding their transgender identity (AOR = 0.57, 95% CI = 0.44–0.98).

**Conclusions:**

Despite the extensive expansion of community-based HIV programs, less than half of transgender women in this national survey had access to the services. Innovative strategies and culturally sensitive interventions should be put in place to reach and respond to the needs of sub-groups of transgender women who are less likely to be reached by the existing traditional approaches.

## Background

According to the World Health Organization (WHO), “transgender people” is an umbrella term for all people whose internal sense of their gender (gender identity) is different from the sex they were assigned at birth [1]. Transgender people choose different terms to describe themselves. There are some transgender people who do not identify themselves as either male or female, but rather outside of a gender binary. In some cultures, specific indigenous terms are used more commonly to describe transgender people or those who identify as a third sex [1]. Transgender women, who are biologically male at birth and self-identify as female [1], are considered at a heightened risk of acquiring HIV and other sexually transmitted infections (STIs) but are strikingly underrepresented in HIV programs and national surveillance systems.

Globally, the prevalence of HIV among transgender women is estimated to be 19.1% (95% CI: 17.4–20.7), which is more than 40 times higher than the prevalence in the general reproductive-age adults [2]. Transgender women experience multiple, intersecting socio-structural risks that increase their vulnerability to HIV, including stigma and discrimination, gender-based violence, lack of social and legal recognition of their affirmed gender, and exclusion from employment and educational opportunities [3, 4]. These situations may expose them to subsequent mental health problems or vice versa [5–7]. Among transgender women living with HIV, these systems of oppression can seriously compromise their sustained access to life-saving treatment and care [3, 8].

The WHO recommends community-based HIV and legal services as a way to reach and respond to the intersecting needs of key populations, including transgender women [9]. This human rights-based approach posits that programs must recognize and respond to the multi-faceted needs arising from transgender women’s social position [4, 8, 10]. It also recognizes that HIV services need to be located within transgender women’s communities or in other sites where they feel comfortable [9, 11, 12]. Furthermore, there has been an increased demand for integrated services that address not only HIV but also other health issues as well as legal and psychosocial needs [12, 13].

In addition to human rights-based arguments, a strong meta-analytic evidence base suggests that community-based HIV services hold promise for improving HIV prevention, treatment, and care. A global systematic review of 117 studies, including 864,651 participants, found that relative to facility-based approaches, community-based programs increased uptake of HIV testing and counselling (HCT), first-time testing, and the proportion of testers with CD4 counts above 350 cells/μl [14]. Another systematic review of 22 cohort studies and randomized control trials, including 97,657 participants, found that community-based HIV programs resulted in comparable ART adherence, virological suppression, and all-cause mortality outcomes as facility-based services [15]. Moreover, community-based programs had the added benefit of higher rates of treatment engagement and cost-saving [4, 15].

However, little is known about the extent to which transgender women engage with community-based programs [16]. Part of the reasons may be a dearth of representative, national-level data on transgender women. Existing data have been collected using inconsistent definitions of transgender, frequently classifying transgender women as a sub-category of men who have sex with men [17]. This approach erases transgender identities, disregards their specific needs and undermines epidemiological data. Moreover, to our knowledge, no studies have examined the health and socio-demographic characteristics of transgender women who access and those who do not access community-based HIV, other health, and legal services.

This paper aims to address these notable gaps using data from a large-scale national bio-behavioral survey of transgender women in Cambodia. This study has two objectives: (1) to examine the extent to which transgender women in Cambodia access community-based HIV and legal services and (2) to identify the characteristics of transgender women who do and do not access community-based HIV services so as to inform targeted services for this underserved population.

## Methods

### Study sites and participants

The National Integrated Biological and Behavioral Survey among transgender women (TG-IBBS 2016) was conducted in the capital city of Phnom Penh and 12 other provinces with high burden of HIV and a large population of transgender women. These provinces were purposively selected by the research team and supported by the national HIV technical working group. More than 70% of the Cambodian population resides in the 13 provinces out of 24 provinces and the capital in Cambodia. According to the national program data from the National Center for HIV/AIDS, Dermatology and STD (NCHADS), in 2015, the selected sites consisted of 21 operational districts (ODs) with the highest-burden of HIV, and more than 80% of men who have sex with men and transgender women in Cambodia.

The Respondent-Driven Sampling (RDS) method was used to recruit participants from 20 specific locations (six locations in Phnom Penh and 14 locations in the remaining 12 provinces). The number of locations was determined based on the estimated population of transgender women and the proportion of the required sample size in each study site. Individuals would be included in the survey if they: (1) were biologically male at birth and self-identified as female, (2) were aged 18 years or older, (3) reported having sex with at least one man in the past 12 months, (4) were able and willing to provide written consent to participate in the study, and (5) were able to communicate in Khmer, the national language of Cambodia. Full details of the methodology have been reported elsewhere [18].

### Training and data collection

A three-day training was provided for all research team members. The training included a review of the study protocol, interview techniques, and confidentiality and privacy protection. It also entailed interview practice through role-play and study procedures through a pilot study. Throughout the data collection, daily review sessions were conducted with interviewers to review progress and communicate any problems that may emerge during the interviews.

Two data collection teams, with eight members each, were formed. Each team included one field supervisor, five interviewers, one counselor, and one lab technician from the Provincial AIDS and STI Program. A peer outreach worker working for transgender women in the area also joined the team as a facilitator. The field supervisor performed participant eligibility screening. A unique personal identification number was assigned to each consenting participant. The number was used to link all data collected from each participant, but it was not linked with any personal identifiers of the participants. Written informed consent was obtained after the counselor informed the participant of the study details. After obtaining the consent, the participant was interviewed in a private room. Each participant received US$4 in cash to compensate for their transport and time and a package of three condoms. In addition, seeds and recruiters received $2 for a successful referral.

#### Variables and measurements

A structured questionnaire was initially developed in English and then translated into Khmer. The Khmer questionnaire was then back-translated by another translator to ensure that the “content and spirit” of every original item was maintained. It was validated through consultative meetings with key stakeholders and representatives of transgender women, pretested with 20 transgender women in Phnom Penh, who were later excluded from the study, and modified based upon comments and suggestions from the pretest and meetings.

Most of the socio-economic and health risk behavior items were adapted from the 2014 Cambodia Demographic and Health Survey [19] and our previous community-based surveys among HIV key populations [20–22]. Socio-demographic characteristics included age, study sites (urban, rural), gender identity, marital status, main occupation, average monthly income in the past six months, completed years of formal education, duration living in the current city, and perceived family economic status. Information regarding gender expression and utilization of gender-affirming hormones and surgeries was also collected. Regarding HIV risks, information collected included sexual behaviors in the past three months such as number of sexual partners, condom use with different types of partners, experiences of STI symptoms, and care seeking behaviors. For transgender women who reported living with HIV, self-reported data on HIV care and treatment were collected.

Regarding access to community-based HIV programs, participants were asked whether they had received the following services in the past three months from a community-based organization: (1) HIV and sexual reproductive health education, (2) condom and lubricant distribution, (3) screening for HIV and STIs, (4) legal support services, (5) other health services such as HIV and STI care and treatment, referral for other health services, etc., and (6) online services developed for transgender women (websites, Facebook pages, hotline calls, etc.). A dichotomous variable was created to capture access to community-based HIV services defined as having been reached by a community-based HIV program through any one of the services in the past three months versus none.

Exposure to gender-based discrimination and violence was measured using items adapted from previous studies [23, 24]. Participants were asked about different forms of discrimination and violence they had experienced. These included whether they felt that their co-workers or classmates were supportive regarding their transgender identity and had experienced problems such as difficulties in getting a job, losing a job, having been denied or thrown out of a housing, and difficulties in getting HIV or other health services and thought it was because of their transgender identity or expression. Participants were asked about their experiences in different forms of violence such as having been physically or sexually abused, been arrested by authorities, dropped out of school, and been fearful of being arrested by police or other authorities because of their transgender identity or expression.

#### Data analyses

Double data entry was performed using EpiData version 3 (Odense, Denmark). STATA Version 12.0 (Stata Corp, Texas, United States) was used to conduct the statistical analyses. All analyses were estimated with sampling weights that corrected for nonresponse and sample design [25]. Bivariate analyses were run, using χ^2^ test (or Fisher’s exact test when a sample size was smaller than five in one cell) for categorical variables and Student’s *t*-test for continuous variables to compare socio-demographic characteristics, HIV risk behaviors, and experiences of gender-based discrimination and violence among transgender women who reported having and having not been reached by community-based HIV programs. To explore independent factors associated with access to community-based HIV programs, a weighted multivariate logistic regression model was constructed, controlling for potential confounders. All variables significantly associated with access to community-based HIV programs in the bivariate analyses at a level of *p-*value < 0.05 were simultaneously included in the model. Age, education level, and income were included regardless of the significance level because of their epidemiological importance. Adjusted odds ratio (AOR) were obtained and presented with 95% confidence interval (CI) and *p*-values.

#### Ethical considerations

The National Ethics Committee for Health Research (NECHR) of the Ministry of Health, Cambodia (No. 420 NECHR) approved the study protocol and materials. A written informed consent was obtained from each participant, and participants were informed that they could decline or withdraw from the study at any time. Privacy and confidentiality were protected by having the interviews conducted in a private room and removing all personal identifiers from all study documents.

## Results

### Community-based HIV programs

The survey sample included 1375 transgender women with a mean age of 25.8 years (SD = 7.1). Table 1 shows the proportion of transgender women living in urban and rural areas who had access to different community-based HIV services in the past three months. Of the total, 45.0% of the participants reported having received at least one community-based HIV service in the past three months. The most common services they had received included condom and lubricant distribution (38.8%), HIV and sexual and reproductive health education (35.6%), online HIV-related information (35.1%), and screening for HIV and STIs (26.9%).

**Table 1:** Community-based HIV programs received by transgender women (*n* = 1375)

HIV services received in the past 3 months	Total	Urban	Rural
	*n* (%)	*n* (%)	*n* (%)
Any community-based HIV services	619 (45.0)	502 (43.8)	117 (51.1)
HIV test in the past 6 months	676 (49.2)	556 (48.5)	120 (52.4)
HIV and sexual reproductive health education	490 (35.6)	402 (35.1)	88 (38.4)
Condoms and lubricants distribution	522 (38.8)	433 (37.8)	89 (38.9)
Screening for sexual transmitted infections	370 (26.9)	304 (26.5)	66 (28.8)
Legal support services	47 (3.4)	40 (3.5)	7 (3.1)
Other health services	110 (8.0)	103 (9.0)	7 (3.1)
Online services for transgender women	483 (35.1)	393 (34.3)	90 (39.3)
Facebook page for transgender women	149 (10.9)	130 (11.3)	19 (8.3)
Website developed for transgender women	96 (7.0)	82 (7.2)	14 (6.1)
Voice messages from or called Voice4U^*^	76 (5.5)	67 (5.8)	9 (3.9)

Abbreviation: HIV, human immunodeficiency virus

*‘Voice4U’ is a mobile phone interactive voice response system that provides callers with online counseling and on-demand HIV and health-related information

### Socio-demographic characteristics

Table 2 shows that the majority of the participants lived in an urban area (83.3%) and were not married or living with a sexual partner (78.1%), and 53.0% were in the age group of 18–24 years. Their median average monthly income in the past six months was US$150 (interquartile range = 90–200), and the mean completed years of formal education they had attained was 9.0 (SD = 3.4). Around one-third (35.1%) were a hairdresser or beautician. Less than half (45.0%) reported having used gender-affirming hormones, and 9.3% having had gender-affirming surgery. Significant differences between participants who reported having and having not been reached by community-based HIV programs were found in type of communities (rural or urban), length of stay in the current location, monthly income, main occupation, frequency of expression as female, number of other transgender women they knew, and use of gender-affirming hormones and surgery.

**Table 2:** Socio-demographic characteristics of transgender women who had and who had no access to community-based HIV programs

Characteristics	Total (*n* = 1375)	Access to community-based HIV programs in the past 3 months^*^		
		No (*n* = 756)	Yes (*n* = 619)	P-value^*^
Living in urban setting	1146 (83.3)	644 (85.2)	502 (81.1)	0.04
Age group				0.07
18–24	729 (53.0)	418 (55.3)	311 (50.2)	
25–34	503 (36.6)	256 (33.9)	247 (39.9)	
≥ 35	143 (10.4)	82 (10.8)	61 (9.9)	
Marital status				0.79
Married	7 (0.5)	4 (0.5)	3 (0.5)	
Widowed/divorced	18 (1.3)	11 (1.5)	7 (1.1)	
Not married/not living with a partner	1070 (78.1)	590 (78.0)	484 (78.2)	
Not married/living with a partner	260 (18.9)	140 (18.5)	120 (18.9)	
Level of formal education attained				0.16
Primary or lower (0–6 years)	307 (22.3)	172 (22.8)	135 (21.8)	
Lower secondary (7–9 years)	440 (32.0)	255 (33.7)	185 (29.9)	
Higher secondary (10–12 years)	503 (36.6)	270 (35.7)	233 (37.6)	
University or higher	125 (9.1)	59 (7.8)	66 (10.7)	
Living in current city ≥16 months	810 (58.9)	430 (56.9)	380 (61.4)	0.09
Average income in the past 6 months ≥US$186	499 (36.3)	259 (34.3)	240 (38.8)	0.08
Current occupation (main source of income)				< 0.001
Unemployed	64 (4.7)	39 (5.2)	25 (4.0)	
Hairdresser/beautician	282 (35.1)	242 (32.0)	240 (38.8)	
Government officer	16 (1.2)	8 (1.1)	8 (1.3)	
Laborer	222 (16.1)	146 (19.3)	76 (12.3)	
Seller	149 (10.8)	80 (10.6)	69 (11.1)	
Entertainment worker	140 (10.2)	78 (10.3)	62 (10.0)	
Sex worker	27 (2.0)	18 (2.4)	9 (1.5)	
Student	108 (7.9)	72 (9.5)	36 (5.8)	
NGO staff	34 (2.5)	4 (0.5)	30 (4.8)	
Private company staff	34 (2.5)	24 (3.2)	10 (1.6)	
Farmer/fisherman	19 (1.4)	9 (1.2)	10 (1.6)	
Artist	36 (2.6)	15 (2.0)	21 (3.4)	
Others	44 (3.2)	21 (2.8)	23 (3.7)	
Frequency of expression as female				< 0.001
All the time	661 (48.1)	331 (43.8)	330 (53.3)	
Not all the time	714 (51.9)	415 (56.1)	289 (46.7)	
Average number of transgender women they knew	23.9 ± 44.6	20.9 ± 36.4	27.7 ± 52.7	0.005
Used gender affirming hormones	619 (45.0)	292 (38.6)	327 (52.8)	< 0.001
Had gender-affirming surgeries	128 (9.3)	57 (7.5)	71 (11.5)	0.01

Values are numbers of subjects (%) for categorical variables and means ± standard deviation (SD) for continuous variables

*Defined by having been reached by a community-based HIV program with any one of the services

^†^Chi-square test (or Fisher’s exact test when a cell count was smaller than 5) was used for categorical variables and independent Student’s t-test for continuous variables

### HIV risk behaviors and HIV testing

According to Table 3, 8.6% of the participants reported having sex with women, and 1.6% reported did so in exchange for money or gifts in the past three months. The majority of respondents (87.5%) reported being in a receptive role in anal intercourse with men, and 81.6% having sex with men not in exchange for money or gifts in the past three months. More than one-third (37.8%) reported having sex with men in exchange for money or gifts, with 60.0% reporting always using condoms with this type of partners. Regarding HIV and STI services, 14.0% had been diagnosed with an STI, and 60.1% tested for HIV in the past six months. Significant differences between the participants who reported having and having not been reached by community-based HIV programs was found in sexual intercourse with women, usual roles in anal sex with men, condom use, and HIV testing.

**Table 3:** HIV risk behaviors and HIV testing among transgender women who had and who had no access to community-based HIV programs

HIV risk behaviors in the past 3 months	Total (*n* = 1375)	Access to community-based HIV programs in the past 3 months^*^		
		No (*n* = 756)	Yes (*n* = 619)	P-value^*†*^
Sex with women	118 (8.6)	76 (10.1)	42 (6.8)	0.03
Sex with women for money or gifts	22 (1.6)	12 (1.6)	10 (1.6)	0.91
Usual role in anal sex with men				0.009
Insertive	29 (2.1)	9 (1.3)	20 (3.3)	
Receptive	1045 (87.5)	626 (88.0)	519 (86.8)	
Both	135 (10.3)	76 (10.7)	59 (9.9)	
Mean number of male partners not in exchange for money or gifts	23.5 ± 39.2	24.1 ± 40.7	22.9 ± 37.4	0.53
Always used condoms with men not in exchange for money or gifts	703 (58.4)	421 (64.7)	282 (51.1)	< 0.001
Sex with men in exchange for money or gifts	495 (37.8)	261 (36.7)	234 (39.1)	0.37
Mean number of male partners in exchange for money or gifts	2.7 ± 10.1	2.6 ± 9.0	2.9 ± 11.3	0.58
Always used condoms with men in exchange for money or gifts	246 (60.0)	112 (52.1)	134 (68.7)	0.001
Had been diagnosed with an STI	193 (14.0)	107 (14.2)	86 (13.9)	0.89
Had HIV test in lifetime	1107 (80.5)	529 (70.0)	578 (93.4)	< 0.001
Had HIV test in the past 6 months	676 (60.1)	212 (28.0)	464 (75.0)	< 0.001
Mean months since last HIV test	8.6 ± 12.3	12.0 ± 14.8	5.4 ± 8.2	< 0.001

Abbreviations: HIV, human immunodeficiency virus; STI, sexually transmitted infections

Values are numbers of subjects (%) for categorical variables and means ± standard deviation (SD) for continuous variables

*Defined by having been reached by a community-based HIV program with any one of the services

^†^Chi-square test (or Fisher’s exact test when a cell count was smaller than 5) was used for categorical variables and independent Student’s t-test for continuous variables

### Gender-based discrimination and violence

It was common for transgender women in this study to experience different forms of discrimination and violence because of their transgender identity (Table 4). Common experiences included difficulties in getting a job (42.0%), sexual abuse or assault (39.1%), feeling fearful of being arrested by police or other authorities (24.9%), having lost a job (24.3%), physical abuse (23.6%), having been denied or thrown out of a housing (18.8%), and feeling that their co-workers or classmates were not supportive regarding their transgender identity (10.1%). Significant difference between participants who reported having been and having not been reached by community-based HIV programs was found in the levels of support from their co-workers or classmates regarding their transgender identity, difficulties in accessing HIV services, physical and sexual abuse and assault, and getting arrested by police or other authorities.

**Table 4:** Gender-based discrimination and violence among transgender women who had and who had no access to community-based HIV programs

Gender-based discrimination and violence	Total (*n* = 1375)	Access to community-based HIV programs in the past 3 months^*^		
		No (*n* = 756)	Yes (*n* = 619)	*P*-value^†^
Co-workers/classmates are supportive regarding transgender identity	1235 (89.8)	667 (88.2)	568 (91.8)	0.03
Difficulties in getting a job	578 (42.0)	302 (39.9)	276 (44.6)	0.08
Lost a job	334 (24.3)	176 (23.3)	158 (25.5)	0.33
Denied/thrown out of a housing	249 (18.1)	127 (16.8)	122 (19.7)	0.16
Having difficulties in getting HIV services	117 (8.5)	48 (6.3)	69 (11.1)	0.002
Having difficulties in getting health services	125 (9.1)	61 (8.1)	64 (10.3)	0.15
Had been physically abused	325 (23.6)	158 (20.9)	167 (27.0)	0.008
Had been sexually abuse or assault	538 (39.1)	267 (35.3)	271 (43.8)	0.001
Had been arrested by police/authorities	144 (10.5)	64 (8.5)	80 (12.9)	0.007
Feeling fearful of being arrested by police	342 (24.9)	180 (23.8)	162 (26.2)	0.31

Values are numbers of subjects (%) for categorical variables

*Defined by having been reached by a community-based HIV program with any one of the services

^†^Chi-square test (or Fisher’s exact test when a cell count was smaller than 5) was used

### Factors associated with access to community-based HIV services

As shown in Table 5, after controlling for potential confounding factors in the multivariate logistic regression model, transgender women who reported having been reached by community-based HIV services remained significantly more likely to reside in an urban setting (AOR = 1.41, 95% CI = 1.01–1.96), have attained university or higher education (AOR = 1.68, 95% CI = 1.02–2.75), have used gender-affirming hormones (AOR = 1.50, 95% CI = 1.17–1.92), report always using condoms with men not in exchange for money or gifts (AOR = 1.81, 95% CI = 0.16–2.82), and have been arrested by police or other authorities because of their transgender identity (AOR = 1.55, 95% CI = 1.03–2.33). Transgender women who reported having been reached by community-based HIV services were significantly less likely to report being in the receptive role (AOR = 0.34, 95% CI = 0.15–0.82) and always using condoms with men not in exchange for money or gifts (AOR = 0.72, 95% CI = 0.55–0.94). Regarding gender-based discrimination and violence, transgender women who reported having been reached by community-based HIV programs were significantly less likely to perceive that their co-workers or classmates were not supportive regarding their transgender identity (AOR = 0.57, 95% CI = 0.44–0.98). Full multivariate logistic regression model including all covariates can be found in Appendix.

**Table 5:** Factors associated with access to community-based HIV programs among transgender women in multivariate logistic regression model (*n* = 1375)

Variables in the final model*	Access to community-based HIV programs^†^	
	AOR (95% CI)	P-value
Study setting		
Rural	Reference	
Urban	1.41 (1.01–1.96)	0.04
Level of formal education attained		
Primary or lower (0–6 years)	Reference	
Lower secondary (7–9 years)	0.96 (0.67–1.32)	0.80
Higher secondary (10–12 years)	1.19 (0.86–1.65)	0.29
University or higher	1.68 (1.02–2.75)	0.04
Use of gender-affirming hormones		
No	Reference	
Yes	1.50 (1.17–1.92)	0.002
Usual role in anal sex with men in past three months		
Insertive	Reference	
Receptive	0.33 (0.14–0.78)	0.01
Both	0.73 (0.31–5.87)	0.09
Always used condoms with men not in exchange for money or gifts		
No	Reference	
Yes	0.67 (0.51–0.87)	0.003
Always used condoms with men in exchange for money or gifts		
No	Reference	
Yes	1.81 (0.16–2.82)	0.009
Feeling that co-workers/classmates are supportive regarding transgender identity		
Supportive	Reference	
Not supportive	0.57 (0.44–0.98)	0.04
Having been arrested by police or authorities because of transgender identity		
No	Reference	
Yes	1.55 (1.03–2.33)	0.03

Abbreviations: AOR, adjusted odds ratio; CI, confidence interval

*Variables in the table were the ones that were statistically significant in the multivariate logistic regression model. Full multivariate logistic regression model including all covariates can be found in Appendix

^†^Defined by having been reached by a community-based HIV program with any one of the services in the past 3 months

## Discussion

One objective of this study is to examine the extent to which transgender women access community-based HIV and legal services in Cambodia. Globally, the needs to offer and improve differentiated services for key populations most affected by HIV, including transgender women, are growing [9]. Findings from this study indicate that almost half of transgender women respondents had been reached by various community-based HIV services to a varying degree. In line with findings from other studies on transgender women and other key populations [14, 15, 26] and the recommendations from the WHO [9], the findings suggest that community-based services are an important gateway to healthcare services and service uptake for transgender women. Besides, access to community-based, gender-affirming, and trans-specific services are known to increase HIV prevention service uptake and to enhance their confidence and involvement in HIV prevention in other studies [23, 27–30]. Compared with other facility-based approaches, community-based programs have been shown to improve HIV prevention and treatment and to be more cost effective [14, 15]. This point was beyond the scope of this research; however, findings from our recent study indicated that transgender women were more likely to use NGO-run and community-based health facilities for HIV testing but to use other health facilities for STI treatment [18]. The effectiveness and efficiency of a health facility, including community-based programs, should thus warrant further investigation.

It is worth-noting that more than half of the respondents in this national survey had not been reached by any community-based HIV programs in the past three months. Besides, among those who accessed the community-based services, the use of legal and other health services (besides HIV and STI) and new media platforms designed specifically to service their needs had been very low. Given the limited access as well as (cost) effectiveness and efficiency of community-based services, more efforts are needed to better understand the needs for community-based intervention programs among transgender women, including exploring reasons why the women are not reached, why they use a facility or not, which services they consume or not, and which approaches are more applicable and yield better impacts. Such information would be useful for designing the interventions to attain optimal results.

This study also offers unprecedented data on the characteristics of transgender women who had and who had not been reached by community-based HIV programs, which is the main and second objective of our study. The findings from the multivariate logistic regression analysis suggest that transgender women who had been reached by community-based HIV programs were more likely to live in urban areas, to use gender-affirming hormones and surgeries, and to have been arrested by police or other authorities due to their transgender identity. They were also more likely to have supportive friends and co-workers of their gender identity and expression. They were less likely to play a receptive role in sexual intercourse and to consistently use condoms with non-commercial male partners [20]. Why and how they possess these characteristics and whether they are associated with or caused by the use of community-based health facilities were not explored in our study and are worth in-depth examination to inform policy formulation and project/program intervention.

The possession of these characteristics by the transgender women is not unique to Cambodia, and many of these traits and practices can be detrimental to their health and well-being. Similar to findings from Thailand, which reported that Thai transgender women had limited access to surgery (11%) [31], this study found that few Cambodian transgender women experienced gender-affirming surgery – which may be linked with their low income as discussed below and the high cost of such surgery. However, a higher number (45%) of transgender women used gender-affirming hormones, and transgender women who had been reached by community-based services were more likely to use such hormones. Inappropriate use of injecting hormones could increase vulnerability to HIV and other blood-borne viruses [31–33]. Our recent study found that the risk of HIV infection was more than four times higher among transgender women who self-injected hormones compared to that among transgender women who did not [34].

That transgender women – whether they access community-based services or not – are likely to be harassed or even arrested by law enforcement officers, particularly for their gender identity and expression, is rather a global phenomenon and has been documented in other studies elsewhere [4, 35]. Together with the fact that they were less likely to receive legal services from community-based organizations and perhaps other facilities too, the alarming situation reinforces the need for comprehensive intervention programs that may include provision of legal services and training and promotion of advocacy skills and capacity [4, 36]. Such programs would require involvement of broader stakeholders at different levels.

A significantly higher proportion of transgender women who had been reached by community-based HIV programs perceived that their co-workers or classmates were supportive regarding their transgender identity. Whether this is a causality or a mere correlation between access to community-based programs and peer support was not covered in the study, and this requires further examination. Apart from the feeling of support as illustrated in the regression model, the findings from our descriptive results show that a fairly high proportion of transgender women reported experiencing different forms of gender-based discrimination and violence. Previous studies in other settings have shown that stigma and discrimination and social exclusion are common among transgender women [37–40]. These may lead to increased unmet needs for healthcare services and hinder their access to prevention programs that in turn extend the vulnerability to HIV infection among this key population. Gender-based discrimination and violence may cause psychological pressure, mental problems or even suicide [37–40]. They are also known to be associated with inconsistent condom use in anal intercourse and sex work among transgender women [41–43].

Our study indicated that transgender women who had been reached by community-based HIV programs were less likely to report always using condoms with non-commercial partners. The finding is inconsistent with results found in a study of transgender women in India, which reported that transgender women who had access to a community-based program were more likely to use condoms with both regular and casual partners [4]. We were not able to account for the difference in this outcome. However, the high rate of inconsistent condom use in different types of relationships among transgender women is a global phenomenon and has been widely documented in other studies, and this was true even among transgender women who had been reached by community-based HIV intervention programs. These practices place them in an increased risk of contracting HIV and other STIs. The inconsistent condom use can partly be explained by the trust in sexual partners, a sense of freedom and intimacy, as well as low self-esteem and power imbalance in condom use negotiation given their gender identity [41, 44, 45].

Two key findings from the descriptive results of the characteristics of transgender women are worth-mentioning. Despite the slightly different income between those who had and who had not been reached by community-based HIV programs, they were more concentrated in the informal economy, which is associated with little social safety net and low pay. The low socio-economic status, such as destitution and job insecurity, among our study participants is in line with the earning and living conditions of their peers elsewhere [23, 27, 28, 41]. This finding suggests that community-based programs may consider including non-HIV related services such as job referral training and placement programs [46] to improve their livelihood and career prospects. Another important point is that the number of transgender women within the network is quite large. While this may have good implications for social and peer support and service uptake, findings from other contexts indicated that such a wide network can have implications for HIV and STI transmission as well as commitment of sex work as a means of livelihood and a choice [31].

Finally, our study confirms the promising use of snowballing and peer-driven intervention for HIV case detection and its implication in HIV prevention, care, and treatment among transgender women. With the use of the Respondent Driven Sampling method, we were able to reach hundreds of transgender women (more than half of the total sample) who never used the community-based HIV services. Peer-driven interventions are known to reach wider targets and to produce better outcomes than a traditional approach in case detection and preventive service uptake and retention in care and treatment [4, 47–49].

Overall, there is a need to better understand transgender women’s access to health and non-health services, especially through the community-based programs, given the limited availability of information and research on this key population and the high prevalence of HIV among this group. Besides, given the (cost) effectiveness, efficiency of community-based programs, the rise of new and social media, and the need for community empowerment and engagement, a holistic approach to HIV prevention and primary care in community-based settings is preferable and can yield better intervention results [26, 50, 51]. Such an integrated approach may consider provision of services beyond HIV and STI prevention, care, and treatment to include other customized services such as job referral, vocational/technical training, financial aid, legal services, psychological support and counseling, and the use of news and social media for health promotion purposes and beyond.

This study has a number of limitations. First, the findings should be interpreted with caution given the cross-sectional nature of the data. The order of effects remains unknown, and it was not meant to determine causality. Further, it focused solely on transgender women in the capital city and 12 provinces with a high burden of HIV and a large population size of transgender women, limiting its nationwide generalizability. More research are needed to examine the needs of transgender women living in more remote and smaller geographical areas believed to have lower burden of HIV. Aside from the HIV testing, all responses relied on self-report measures that are prone to recall and social desirability response bias.

## Conclusions

We found that, despite substantial investments in community-based HIV prevention programs, access to these programs among transgender women in Cambodia remains suboptimal. Only less than half of the women in the study had access to some forms of community-based HIV and legal support services provided by NGOs. To strengthen the response to the needs of this key and vulnerable population, community-based intervention programs should be tailored to respond to the needs of sub-groups of transgender women who are less likely to access the traditional outreach approaches. Using the Respondent Driven Sampling method, our study could reach more than half of participants who had never been reached by any community-based HIV programs. Therefore, a snowball approach could be an effective strategy to reach transgender women who had not been reached by NGOs in the communities.

## Appendix

**Table 6:** Variables included in multivariate logistic regression model (*n* = 1375)

Variables in the final model*	Access to community-based HIV programs^†^	
	AOR (95% CI)	P-value
Study setting		
Rural	Reference	
Urban	1.41 (1.01–1.96)	0.04
Age group		
18–24	Reference	
25–34	1.12 (0.86–1.44)	0.41
≥ 35	0.85 (0.57–1.27)	0.42
Level of formal education attained		
Primary or lower (0–6 years)	Reference	
Lower secondary (7–9 years)	0.96 (0.67–1.32)	0.80
Higher secondary (10–12 years)	1.19 (0.86–1.65)	0.29
University or higher	1.68 (1.02–2.75)	0.04
Average monthly income in the past six months		
< US$186	Reference	
≥ US$186	1.02 (0.79–1.30)	0.90
Current occupation (main source of income)		
Entertainment worker	Reference	
Hairdresser/beautician	0.99 (0.70–1.42)	0.97
Government officer	1.45 (0.82–2.58)	0.20
Laborer/farmer/fisherman	0.80 (0.53–1.21)	0.29
Self-employed	1.13 (0.72–1.78)	0.60
Unemployed	0.81 (0.44–1.50)	0.51
Student	0.67 (0.38–1.17)	0.16
Other	1.07 (0.54–2.14)	0.85
Duration living in current city		
< 16 months	Reference	
≥ 16 months	1.17 (0.93–1.48)	1.18
Frequency of expression as female		
All the time	Reference	
Not all the time	0.89 (0.69–1.16)	0.40
Use of gender-affirming hormones		
No	Reference	
Yes	1.50 (1.17–1.92)	0.002
Had gender-affirming surgeries		
No	Reference	
Yes	1.05 (0.70–1.59)	0.81
Had sex with women in the past 12 months		
No	Reference	
Yes	1.06 (0.48–2.35)	0.89
Usual role in anal sex with men in past three months		
Insertive	Reference	
Receptive	0.33 (0.14–0.78)	0.01
Both	0.73 (0.31–5.87)	0.09
Always used condoms with men not in exchange for money or gifts		
No	Reference	
Yes	0.67 (0.51–0.87)	0.003
Always used condoms with men in exchange for money or gifts		
No	Reference	
Yes	1.81 (0.16–2.82)	0.009
Feeling that co-workers/classmates are supportive regarding transgender identity		
Supportive	Reference	
Not supportive	0.57 (0.44–0.98)	0.04
Having been arrested by police or authorities because of transgender identity		
No	Reference	
Yes	1.55 (1.03–2.33)	0.03
Having difficulties in getting HIV services		
No	Reference	
Yes	1.07 (0.71–1.60)	0.76
Had been physically abused		
No	Reference	
Yes	1.27 (0.96–1.69)	0.10
Had been sexually abuse or assault		
No	Reference	
Yes	1.12 (0.93–1.53)	0.16
Had been arrested by police/authorities		
No	Reference	
Yes	1.36 (0.91–2.02)	0.13

Abbreviations: AOR, adjusted odds ratio; CI, confidence interval

*Age, education level, and income and all variables significantly associated with access to community-based HIV programs in the bivariate analyses at a level of p-value < 0.05 were simultaneously included in the model

^†^Defined by having been reached by a community-based HIV program with any one of the services in the past 3 months

## Authors’ information

SY (PhD) is Senior Research Fellow at Saw Swee Hock School of Public Health, National University of Singapore, Singapore and Director of KHANA Center for Population Health Research, Cambodia, SS (PhD) is Senior Research Fellow at KHANA Center for Population Health Research. ST (MA) is Manager,; PC (MPH) is Research Fellow; and SS (PhD) is Senior Advisor at KHANA Center for Population Health Research, Cambodia. SC (MHSc) is Senior Research Officer at FHI 360, Cambodia; PM (MPH) is Chief and NC (MPH) is Deputy Chief of Surveillance Unit, National Center for HIV/AIDS, Dermatology and STD; MP (PhD) is Senior Advisor for Research and Evaluation at Frontline AIDS, the United Kingdom.

## References

[1] World Health Organization (2015). Policy Brief: Transgender People and HIV.

[2] Baral SD, Poteat T, Strömdahl S, Wirtz AL, Guadamuz TE, Beyrer C (2013). Worldwide burden of HIV in transgender women : a systematic review and meta-analysis. Lancet.

[3] Poteat T, Reisner SL, Radix A (2014). HIV epidemics among transgender women. Curr Opin HIV AIDS.

[4] Shaikh S, Mburu G, Arumugam V, Mattipalli N, Aher A, Mehta S (2016). Empowering communities and strengthening systems to improve transgender health: outcomes from the programme in India. J Int AIDS Soc.

[5] Benotsch EG, Zimmerman R, Cathers L, McNulty S, Pierce J, Heck T (2013). Non-medical use of prescription drugs, polysubstance use, and mental health in transgender adults. Drug Alcohol Depend.

[6] Chakrapani V, Newman PA, Shunmugam M, Logie CH, Samuel M (2017). Syndemics of depression, alcohol use, and victimisation, and their association with HIV-related sexual risk among men who have sex with men and transgender women in India. Glob Public Health..

[7] Yi S, Tuot S, Chhim S, Chhoun P, Mun P, Mburu G (2018). Exposure to gender-based violence and depressive symptoms among transgender women in Cambodia: findings from the National Integrated Biological and behavioral survey 2016. Int J Ment Health Syst.

[8] Lacombe-Duncan A (2016). An intersectional perspective on access to HIV-related healthcare for transgender women. Transgend Health.

[9] World Health Organization (WHO) (2017). Key considerations for differentiated antiretroviral therapy delivery for specific populations: children, adolescents, pregnant and breastfeeding women and key populations.

[10] Reisner SL, Poteat T, Keatley J, Cabral M, Mothopeng T, Dunham E (2016). Global health burden and needs of transgender populations: a review. Lancet.

[11] International HIV/AIDS Alliance (2010). Greater involvement of people living with HIV (GIPA): good practice guide.

[12] International HIV/AIDS Alliance (2017). Putting people at the heart of the HIV response.

[13] Macdonald V, Verster A, Baggaley R (2017). A call for differentiated approaches to delivering HIV services to key populations. J Int AIDS Soc.

[14] Suthar AB, Ford N, Bachanas PJ, Wong VJ, Rajan JS, Saltzman AK (2013). Towards universal voluntary HIV testing and counselling: a systematic review and meta-analysis of community-based approaches. PLoS Med.

[15] Nachega JB, Adetokunboh O, Uthman OA, Knowlton AW, Altice FL, Schechter M (2016). Community-based interventions to improve and sustain antiretroviral therapy adherence, retention in HIV care and clinical outcomes in low- and middle-income countries for achieving the UNAIDS 90-90-90 targets. Curr HIV/AIDS Rep.

[16] Baral S, Holland CE, Shannon K, Logie C, Semugoma P, Sithole B (2014). Enhancing benefits or increasing harms: community responses for HIV among men who have sex with men, transgender women, female sex workers, and people who inject drugs. J Acquir Immune Defic Syndr.

[17] Kaplan RL, Sevelius J, Ribeiro K (2016). In the name of brevity: the problem with binary HIV risk categories. Glob Public Health.

[18] Yi S, Ngin C, Tuot S, Chhoun P, Chhim S, Pal K (2017). HIV prevalence, risky behaviors, and discrimination experiences among transgender women in Cambodia: descriptive findings from a national integrated biological and behavioral survey. BMC Int Health Hum Rights.

[19] National Institute of Statistics (2014). Directorate general for health, and ORC macro. Cambodia demographic and health survey 2014.

[20] Weissman A, Ngak S, Srean C, Sansothy N, Mills S, Ferradini L (2016). HIV prevalence and risks associated with HIV infection among transgender individuals in Cambodia. PLoS One.

[21] Yi S, Tuot S, Chhoun P, Brody C, Tith K, Oum S (2015). The impact of a community-based HIV and sexual reproductive health program on sexual and healthcare-seeking behaviors of female entertainment workers in Cambodia. BMC Infect Dis.

[22] Yi S, Tuot S, Chhoun P, Pal K, Ngin C, Choub SC, Brody C (2016). Improving prevention and care for HIV and sexually transmitted infections among men who have sex with men in Cambodia: the sustainable action against HIV and AIDS in communities (SAHACOM). BMC Health Serv Res.

[23] Clements-Nolle K, Marx R, Guzman R, Katz M (2001). HIV prevalence, risk behaviors, health care use, and mental health status of transgender persons: implications for public health intervention. Am J Public Health.

[24] Early MS (2016). Gender discrimination on health outcomes and behaviors among African-American transgender women in Atlanta.

[25] Bell BA, Onwuegbuzie AJ, Ferron JM, Jiao QG, Hibbard ST, Kromrey JD (2012). Use of design effects and sample weights in complex health survey data: a review of published articles using data from 3 commonly used adolescent health surveys. Am J Public Health.

[26] Melendez RM, Pinto RM (2009). HIV prevention and primary care for transgender women in a community-based clinic. J Assoc Nurses AIDS Care.

[27] Reback CJ, Ferlito D, Kisler KA, Fletcher JB (2015). Recruiting, linking, and retaining high-risk transgender women into HIV prevention and care services: an overview of barriers, strategies, and lessons learned. Int J Transgend.

[28] Pinto RM, Melendez RM, Spector AY. Male-to-female transgender individuals building social support and capital from within a gender-focused network. J Gay Lesbian Soc Serv 2008;20(3):203–20. 10.1080/10538720802235179 PMC2858872 20418965

[29] Reisner SL, Bradford J, Hopwood R, Gonzalez A, Makadon H, Todisco D, et al. Comprehensive transgender healthcare: the gender affirming clinical and public health model of Fenway health. J Urban Health. 2015;92(3):584–92. 10.1007/s11524-015-9947-2 PMC4456472 25779756

[30] Center for Excellence for Transgender Health (2012). 8 best practices for HIV prevention among trans people.

[31] Guadamuz TE, Wimonsate W, Varangrat A, Phanuphak P, Jommaroeng R, McNicholl JM (2011). HIV prevalence, risk behavior, hormone use and surgical history among transgender persons in Thailand. AIDS Behav.

[32] Poteat T, Scheim A, Xavier J, Reisner S, Baral S (2016). Global epidemiology of HIV infection and related Syndemics affecting transgender people. J Acquir Immune Defic Syndr.

[33] World Health Organization (2011). Guidelines for the prevention and treatment of HIV and other sexually transmitted infections among men who have sex with men and transgender people: recommendations for a public health approach 2011.

[34] Chhim S, Ngin C, Chhoun P, Tuot S, Ly C, Mun P (2017). HIV prevalence and factors associated with HIV infection among transgender women in Cambodia: results from a national integrated biological and behavioral survey. BMJ Open.

[35] Schneiders ML, Weissman A (2016). Determining barriers to creating an enabling environment in Cambodia: results from a baseline study with key populations and police. J Int AIDS Soc.

[36] Neumann MS, Finlayson TJ, Pitts NL, Keatley J (2017). Comprehensive HIV prevention for transgender persons. Am J Public Health.

[37] Delany-Moretlwe S, Cowan FM, Busza J, Bolton-Moore C, Kelley K, Fairlie L (2015). Providing comprehensive health services for young key populations: needs, barriers and gaps. J Int AIDS Soc.

[38] Lall P, Lim SH, Khairuddin N, Kamarulzaman A (2015). An urgent need for research on factors impacting adherence to and retention in care among HIV-positive youth and adolescents from key populations. J Int AIDS Soc.

[39] Brennan J, Kuhns LM, Johnson AK, Belzer M, Wilson EC, Garofalo R (2012). Syndemic theory and HIV-related risk among young transgender women: the role of multiple, co-occurring health problems and social marginalization. Am J Public Health.

[40] Reisner SL, White JM, Bradford JB, Mimiaga MJ (2014). Transgender health disparities: comparing full cohort and nested matched-pair study designs in a community health center. LGBT health.

[41] De Santis JP (2009). HIV infection risk factors among male-to-female transgender persons: a review of the literature. J Assoc Nurses AIDS Care.

[42] Stahlman S, Liestman B, Ketende S, Kouanda S, Ky-Zerbo O, Lougue M (2016). Characterizing the HIV risks and potential pathways to HIV infection among transgender women in cote d’Ivoire, Togo and Burkina Faso. J Int AIDS Soc.

[43] de Lind van Wijngaarden JW, Schunter BT, Iqbal Q (2013). Sexual abuse, social stigma and HIV vulnerability among young feminised men in Lahore and Karachi. Pakistan Cult Health Sex.

[44] Baral S, Beyrer C, Muessig K, Poteat T, Wirtz AL, Decker MR (2012). Burden of HIV among female sex workers in low-income and middle-income countries: a systematic review and meta-analysis. Lancet Infect Dis.

[45] Schunter BT, Cheng WS, Kendall M, Marais H (2014). Lessons learned from a review of interventions for adolescent and young key populations in Asia Pacific and opportunities for programming. J Acquir Immune Defic Syndr.

[46] Schulden JD, Song B, Barros A, Mares-DelGrasso A, Martin CW, Ramirez R (2008). Rapid HIV testing in transgender communities by community-based organizations in three cities. Public Health Rep.

[47] UNAIDS (2016). Joint United Nations Programme on HIV/AIDS. Prevention gap report.

[48] Lowe D, Hales D, Burns K, Pick B, Cassell M, Mao B (2016). Evaluation of the USAID/Cambodia PEPFAR HIV/AIDS flagship project. Phnom Penh: KHANA.

[49] Annual KHANA (2017). Progress report to USAID/Cambodia (HIV/AIDS flagship).

[50] Reisner SL, Mimiaga MJ, Bland S, Mayer KH, Perkovich B, Safren SA (2009). HIV risk and social networks among male-to-female transgender sex workers in Boston, Massachusetts. J Assoc Nurses AIDS Care..

[51] Nemoto T, Operario D, Keatley J, Nguyen H, Sugano E (2005). Promoting health for transgender women: transgender resources and neighborhood space (TRANS) program in San Francisco. Am J Public Health.

